# Two birds with one stone: experiences of combining clinical and research training in addiction medicine

**DOI:** 10.1186/s12909-017-0862-y

**Published:** 2017-01-23

**Authors:** J. Klimas, R. McNeil, K. Ahamad, A. Mead, L. Rieb, W. Cullen, E. Wood, W. Small

**Affiliations:** 1 British Columbia Centre for Excellence in HIV/AIDS, St. Paul’s Hospital, 608-1081 Burrard Street, Vancouver, BC V6Z 1Y6 Canada; 2 Department of Medicine, University of British Columbia, St. Paul’s Hospital, 608-1081 Burrard Street, Vancouver, BC V6Z 1Y6 Canada; 3 School of Medicine, University College Dublin, Belfield, Dublin 4, Ireland; 4 Department of Family Practice, University of British Columbia, St. Paul’s Hospital, Vancouver, BC Canada; 5 Department of Family and Community Medicine, 1081 Burrard St, Vancouver, BC V6Z 1Y6 Canada; 6 Faculty of Health Sciences, Simon Fraser University, Burnaby, Canada; 7 Urban Health Research Initiative, B.C. Centre for Excellence in HIV/AIDS, University of British Columbia, St. Paul’s Hospital, 608-1081 Burrard Street, Vancouver, BC V6Z 1Y6 Canada

**Keywords:** Clinician-scientist, Substance-related disorders, Medical education, Qualitative research

## Abstract

**Background:**

Despite a large evidence-base upon which to base clinical practice, most health systems have not combined the training of healthcare providers in addiction medicine and research. As such, addiction care is often lacking, or not based on evidence or best practices. We undertook a qualitative study to assess the experiences of physicians who completed a clinician-scientist training programme in addiction medicine within a hospital setting.

**Methods:**

We interviewed physicians from the St. Paul’s Hospital Goldcorp Addiction Medicine Fellowship and learners from the hospital’s academic Addiction Medicine Consult Team in Vancouver, Canada (*N* = 26). They included psychiatrists, internal medicine and family medicine physicians, faculty, mentors, medical students and residents. All received both addiction medicine and research training. Drawing on Kirkpatrick’s model of evaluating training programmes, we analysed the interviews thematically using qualitative data analysis software (Nvivo 10).

**Results:**

We identified five themes relating to learning experience that were influential: (i) attitude, (ii) knowledge, (iii) skill, (iv) behaviour and (v) patient outcome. The presence of a supportive learning environment, flexibility in time lines, highly structured rotations, and clear guidance regarding development of research products facilitated clinician-scientist training. Competing priorities, including clinical and family responsibilities, hindered training.

**Conclusions:**

Combined training in addiction medicine and research is feasible and acceptable for current doctors and physicians in training. However, there are important barriers to overcome and improved understanding of the experience of addiction physicians in the clinician-scientist track is required to improve curricula and research productivity.

## Background

Socio-structural factors continue to drive problematic drug use and related harms. A lack of physician training in addiction medicine partially shapes the barriers to proper care and treatment for people who use drugs (PWUD). Although it has been argued that there has been too much focus on information provision as a route toward behaviour change, addressing deficits in physician training represents an opportunity to employ educational approaches to improve health of PWUD. Advances in addiction science have helped to identify effective treatments, in particular early identification and treatment of substance-related disorders [21]. Unfortunately, these treatments are under-utilized by physicians, in part due to a lack of knowledge and accredited training programmes in addiction medicine [34, 47].

Among the few programmes recognised internationally is the Dutch two-year Master in Addiction Medicine (MiAM) integrates clinical-practice learning with theory on evidence-based medicine, communication and basic psychotherapeutic skills, neurobiology of addiction, addiction medicine, addiction and psychiatry, and public health, thus shaping seven core competencies of future addiction professionals [9]. Similarly, Norway has created a full medical specialty in addiction medicine in response to the government’s mandate from 2010. The first cohort was trained in Autumn 2014 [20]. The new Australian training scheme, modelled on the internal medicine training programme, offers three years of discipline-specific supervised training with continuous assessment and broad focus on harm reduction and evidence-based treatment [18]. Given the scarcity of existing training programmes, evaluation research is warranted to better understand experience of learners in these programmes and guide future training efforts.

Clinician-scientists bridge the gap between addiction research and clinical practice. Their capacity to translate research into practice is greater given their understanding of human disease and people, as both patients and as research subjects [5]. Non-researcher clinicians and non-clinician researchers do not have this advantage [30]. To this end, the literature has demonstrated how low rates of research training among physicians (in areas other than addiction) are attributable to a range of factors including debt associated with medical education and inflexible educational programmes [10, 19, 41, 51].

Concerns persist about the feasibility of combining clinical and research training in addiction medicine. Combined training is impeded by the inflexibility of standard training programmes, as well as the lack of hands-on experience, mentors, career counselling and supportive training environments [3, 43, 48, 49]. Furthermore, established physicians often do not receive formal recognition for additional research training and face competing clinical priorities stemming from patient care [19, 29, 49].

Recently, the St. Paul’s Hospital Goldcorp Addiction Medicine Fellowship [53], and a Research Fellowship in addiction medicine funded by the U.S. National Institute of Drug Abuse, have been established in Vancouver, Canada, to address these challenges [40]. These fellowships provide support from researchers, statisticians, and the hospital Addiction Medicine Consult Team to fellows, medical students and residents on rotations to facilitate research training and experience. This type of learning environment is rare, and examination of learners’ experiences is merited. Knowledge of their experiences would help us better understand their needs and tailor educational programmes accordingly. We conducted a qualitative study of learners’ experiences of combined clinical and research training in addiction medicine.

## Methods

Qualitative interviews were conducted to examine the experiences of combined clinician-scientist training in addiction medicine through the two Fellowships.

### Setting

The St. Paul’s Hospital Goldcorp Addiction Medicine Fellowship and the Canada Addiction Medicine Research Fellowship provide physicians who have completed foundational clinical training in family medicine, psychiatry, internal medicine, or other speciality with further training in addiction medicine. While the focus of the clinical fellowship is on direct patient care, both streams receive research training. The core learning occurs within the Addiction Medicine Consult Team (AMCT) at St. Paul’s Hospital that provides inpatient Addiction Medicine consultations [40]. Consultations involve addiction treatment coordination for medical co-morbidities, opioid agonist therapy initiation, continuation and monitoring, as well as inpatient withdrawal and detoxification management, and motivational interviewing. The team conducts all follow-up treatment recommendations and coordination. Addiction Medicine Fellows, medical students, residents and enhanced skills learners are accepted for rotations. The Fellows perform senior tasks and share team oversight with the AMCT members. The team and the Principal Investigator (PI) provide comprehensive research training. Learners’ obtain data from Urban Health Research Initiative’s (UHRI) cohort studies of HIV-negative injection drug users, HIV-positive drug users, and street-involved youth, described elsewhere [22, 46, 52], and receive statistical support from statisticians to develop analyses for publication [25].

### Recruitment

Potential participants included all learners who had completed the clinical fellowship, research fellowship or enhanced skills training; the staff of the AMCT; and any students or residents who have done a one-month rotation and also prepared a research publication. Also the teaching faculty for the fellowship (including nurses, social workers and the fellowship director) were invited to participate in the qualitative interviews to better understand training processes and objectives. The participants were invited via email by the principal investigator, followed by reminders from an assistant; 26 (84%) of the 2013–15 cohort members participated.

### Data collection

Interviews utilized a topic guide informed by a scoping review of the literature and a previous qualitative study on a similar topic with health professionals in Portland, Oregon [27]. The topic guide made use of a series of questions focused on the experience of learning, positive and negative aspects of the training, and the training environment. Interviews were audio-recorded and transcribed verbatim. Interviews started with a journey plotting exercise, which was used to capture the experience of learning and related emotion over time [33]. At the beginning of the interview, we asked participants to draw a timeline of their training (five interviews were done over phone, without journey plots). They marked significant events and corresponding high and low points. The horizontal axis represented time and the vertical axis described emotional experience. Journey plots are well suited to capturing experiences and corresponding emotions through time [33, 35]. We asked interviewees to note the significant milestones in their learning experiences and to comment on them. This way, we collected empirical data regarding the learning experience, as well as the related meaning or emotional dimension. While the quantification of research productivity via the number of published papers (i.e., track record), is the most frequently used evaluation indicator, it is limited and a richer, more complete evaluation of the research-training process is needed [31]. An in-depth understanding of the training process would help us better tailor educational programmes to the specific needs of learners [41, 49]. We chose a qualitative research design because it can help uncover the meaning and characteristics of learning experience not easily captured with quantitative research approaches [36, 44].

### Data analysis

Five steps guided a qualitative analysis of interview transcripts: 1) data preparation, transcription and familiarization, 2) generation of initial codes, 3) theme assessment, 4) theme review, and 5) theme finalization [4, 12]. Curran and Fleet’s adaptation of Kirkpatrick’s model was used to conceptualise our analytical framework in relation to four levels of evaluating training programmes: 1) learner satisfaction, 2) learning outcomes (i.e., skills, knowledge and attitudes), 3) performance improvement (i.e., behaviour) and 4) patient outcomes [8]. The first author generated themes based upon a priori and emergent codes, and used qualitative data analysis software (NVivo 10, www.qsrinternational.com) to code transcript material. Two external reviewers examined selected quotes, considering them against the themes. Our analysis integrated the interview and the journey plot findings. We looked for similarities and differences in the journey-plot curves and grouped similar plots together. We sought to understand the patterns in learning experiences by identifying the tempo (how quickly learning went) and the intensity (how significant the learning was) of learning.

## Results

### Socio-demographic characteristics

Our participants included clinical fellows (*n* = 8), research fellows (*n* = 4) or enhanced skills learners (*n* = 2), and all students or residents who have done a one-month rotation and also prepared a research report (*n* = 11); as well as the staff of the AMCT and teaching faculty for the fellowship (including nurse, social worker and fellowship ex-director, *n* = 4). Participant numbers do not add up to 26 because some participants fell into several categories. Their mean age was 33 years (range 25–53 years) and 14 were women.

### Thematic analysis

Using Kirkpatrick’s conceptual model, thematic analysis revealed five major themes relating to learning experience which were influential: (i) attitude, (ii) knowledge, (iii) skill, (iv) behaviour and (v) patient outcome. The presence of a supportive learning environment, flexibility in time lines, highly structured rotations, and clear guidance regarding manuscript development helped facilitate clinician-scientist training. Competing priorities, including clinical and family responsibilities, hindered training.

### Attitude towards people with addictions

In line with the vision for the fellowship, trainees reported acquiring better advocacy skills and more empathic disposition as a product of the training:

I think I’ve learned more about advocacy, and because I had the chance to spend a whole dedicated year to like “addiction”. I had a chance to think about all the socioeconomic factors, I went to all the different rotations …I was, all the different people at different points [in varying rotations].” [Participant #24, Female, 30 years old]

The participants acted in multiple different roles through the rotations. Improved empathy among trainees was reported to be a product of interactions with individual mentors, rather than a function of the training programme itself:

“Cause it’s nice to get the letters [clinical designation], but I think the main aim is to have compassionate clinicians who treat people with addiction well.” [Participant #12, Female, 34 years]

Longer clinical rotations similarly supported the development of empathy among trainees, and some felt empathy is in fact a characteristic that can be taught:

“I think often there’s a belief that you can’t teach someone empathy but you really can, and I see that in the residents who come for electives with me, especially if they come for a long time.” [#12, Female, 34 years]

### Improved knowledge of addiction science and addiction treatment

Overall, the learners described how the fellowship produced “heightened awareness” of addiction medicine, and addressed the deficiencies in addiction-related education from their previous medical training. Addiction-related education in medical school was largely perceived as inadequate:

“I didn’t go to medical school here so I’m not sure how it’s structured here but we definitely have clinical skills and communications curricula that the cases where you would have a respirology case, and a cardiology case and a haematology case, and why not have an addictions case weaved into that [#16, Female, 27 years]

Further, trainees’ knowledge of the limited addiction-specific coursework and content was not regularly tested in standard training:

“they test, you know, 5% of the course. 95% is untested- so it doesn’t encourage people to go to it because they don’t really care too much.” [#6, Male, 27 years]

As a product of the fellowship, participants recognised addiction as a disease with all its social and medical dimensions, as well as the related public-health, harm-reduction and evidence-based responses:

“I think the fellowship really was geared to teaching the science behind addiction and the science behind the management of addiction.” [#10, Female, 34 years]

Faculty had a similar perspective, reporting that training Fellows keeps mentors and teachers current regarding best practices and treatments:

“being part of the clinical fellowship helps to really keep you on your toes.” [#9, Female, 29 years]

### Improving clinical skills

It was reported that clinical, research and generic skills (i.e., communication, teamwork, etc.) improved due to training received through the Fellowship. Clinically, participants felt the training was highly beneficial, providing extensive opportunities to interact with people who had addictions issues:

“[it was] good because we saw a lot of very sick patients, so just like learning how to diffuse situations, and how to go around asking the information that you need to know, and how to observe patients while you’re talking to them.” [#17, Female, 25 years]

Overall, the high proportion of patients with addiction issues present within the hospital environment where training was provided was perceived to be conducive to learning, but this could be challenging, at times. While trainees sometimes experienced difficulties conducting patient exams, interactions with people with severe addictions provided opportunities to navigate past these challenges:

“I think the clientele here definitely affects the type of learning and then it’s hard to learn how to take proper history and full physical examinations here, as opposed to somewhere more remote. I find that some of the patients are not as compliant, or not as accepting of students, at times, and they sometimes just hate medical students or doctors, in general [laughter], so I mean that happens everywhere, but I’ve had more incidents where a patient would just be […] less accepting.” [#18, Male, 26 years]

### Improving research skills

Fellows reported that the flexibility, structure and clear guidance provided within the Fellowship training facilitated the acquisition of research skills. In addition, the mixture of guided and independent learning was seen to be beneficial:

“so he [my mentor] let me pick an area that interested me. He gave a lot of oversight at each stage of the process, like developing the research question, doing the data request, getting a sense of how to interpret the stats, and then writing it up. So, he was very involved at each stage of the way, but I still somewhat independently gave it a shot the first time around, and so it raised my confidence.” [#25, Female, 32 years]

Similarly, autonomy in research training was seen to be strength of the Fellowship:

“I think you’re provided with a lot of autonomy, which I like.” [#10, Female, 34 years]

Competing priorities hindered clinician-scientist training, particularly balancing research tasks with the demands of clinical work and rotations:

“I find that a lot of people who are engaged in the research process really like it. It’s their primary focus, and so I often feel guilty for still really wanting to be involved as a clinician. And so I kind of see it fifty: fifty almost, but that’s really difficult to do.” [#10, Female, 34 years]

Achieving balance between clinical work and research tasks was difficult:

“It’s a tough balance, I think, between being a clinician and trying to be a researcher at the same time [#1, Male, 43 years]”.

### Performance improvement: behaviour

The learners felt that they became better health professionals as a result of the experiences and training:

“when you step into the addiction medicine world, all of a sudden even your relationship with your patients changes a little bit, so I find that the way I behave and my professional demeanour around my patients has changed a bit throughout this year.” [#20, Male, 29 years]

Similarly, Faculty felt that participation, as a mentor, was also beneficial for their clinical practice the same way:

“Yeah, I’m a better doctor because I’m a teacher at the fellowship, I think.”[#12, Female, 34 years]

### Performance improvement: patient outcome

Fewer participants reported pronounced changes but their comments indicate recognition that flexibility regarding patients’ goals was paramount:

“These patients have very different needs than other patients. You need to be a lot more flexible and I think you have to; I guess it all just comes down to re-prioritizing what you want your outcomes to be, but [prioritizing] their out[comes]- what outcomes *they* want.” [#21, Male, 27 years]

The Fellowship helped develop a patient-centred focus. This focus became evident when the fellowship changed from offering short-term rotations exclusively, to include some long-term, rotations, e.g., following patients longitudinally. Initially, the fellows reported that short rotations did not fully maximize learning opportunities:

“I think we as a fellowship has been fairly universal in our feedback about this rotation, that we didn’t get enough longitudinal stuff in there.” [#15, Male, 35 years]

### Findings of analysis of the journey plots

**Fig. 1:**
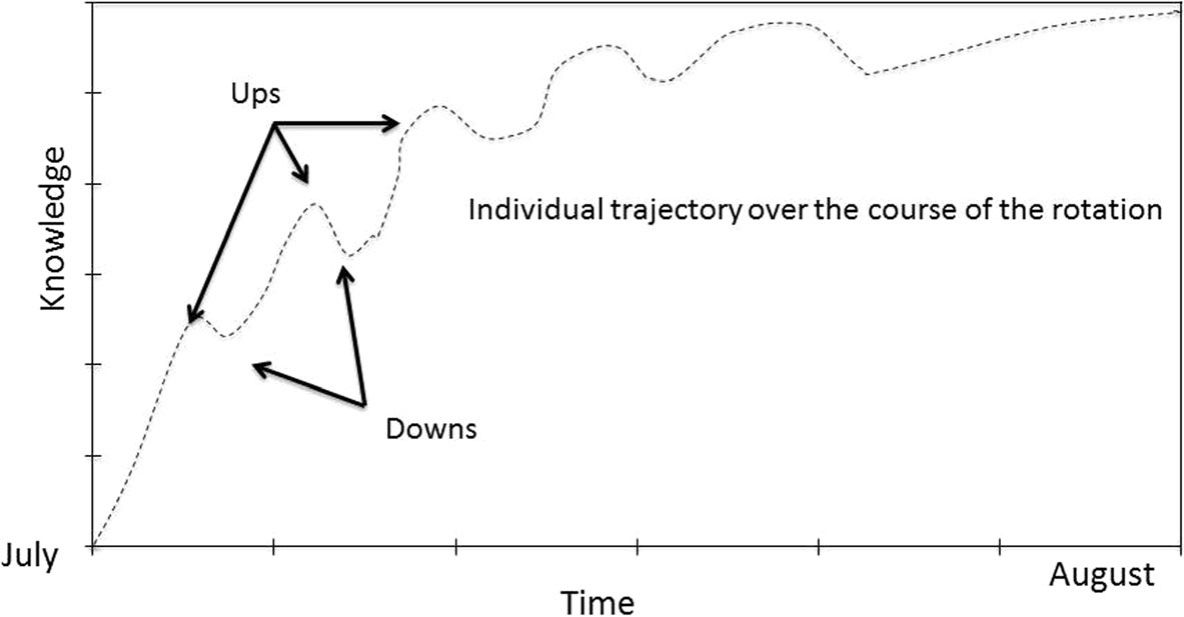
Sample journey plot depicting experience of a resident in the combined clinician-scientist training programme. This sample journey plot depicts the experience of participant #3 during a first rotation as a senior resident. ^a^Downs: “day in and day out and going to work, there are certain you know certain times where you feel like you’re having a day off […] there are ups and downs with sometimes not be[−ing] able to connect or establish a rapport with somebody”. ^b^Ups: “i do remember a lot of really nice moments; a lot of people who i think i was able to help establish a nice plan for their addiction that would help them actually get out into the community, into a supported living environment that required them to be on a certain kind of methadone, or a certain kind of treatment”

Within the 21 journey plots there was a general pattern evident in approximately half of the learners’ journeys: many highs and lows above and below the neutral point with the final point high up (Fig. 1). Put simply, the journey was relatively high paced (speed of learning) and high strength (emotional intensity of experiences):

“I felt there was a very steep learning curve and I had very little background or knowledge in the area and initially, I felt not overwhelmed, but I had a lot to learn and then, as the year went on, that decreased as I started to get more exposure and get more comfortable with the patient population.” [#10, Female, 34 years]

In contrast, seven participants drew a flat line indicating a smooth, relatively eventless learning, as illustrated in the following quote of a person who was a learner and subsequently has taught fellows:

“[…] it has been wonderful [training], I love teaching the fellows, I love working with the fellows that have graduated, it’s so collegial and pleasant.” [#12, Female, 34 years]

Five participants described a linear upward trend (with slow pace) and very small dips (low strength) indicated by 3–5 significant events:

“Clinically I feel like it was always just going up and up and up and up. It was different than being a resident in that I know now how to function within the medical system so clinically I didn’t find it difficult.”[#19, Female, 35 years]

For two participants, many ups and downs (relatively slow pace) oscillating high and low around the neutral point (low strength) and showing mild emotions were illustrated by descriptions of many significant events:

“It was really a steep learning curve for me because there was lots to do, and working as part of a team […] even though that was a good experience, it was a hard experience- so maybe I draw the line kind of up and down at that point for a little bit, almost like a squiggly line.” [#22, Male, 33 years]

One participant experienced an unusually low pace; the strength was high, with only one huge dip shortly after the start, followed by an upward trend. He explained:

“it was around then [beginning of the training, points to the dip in the journey plot] when things sort of started to kind of fall apart a little bit. I think one of the biggest road blocks was just getting the case [study data] […] actually getting access to the case, to the chart took [a very long time].” [#7, Male, 25 years]

While experiences like this were rare, it points to the importance of support, supervision and monitoring in addiction medicine education.

## Discussion

Analysis of qualitative interviews explored the experience of training in addiction medicine and research. The presence of a supportive learning environment, flexibility in time lines, structured rotations, and clear guidance regarding research product development helped facilitate clinician-scientist training. Competing priorities, including clinical and family responsibilities, hindered training. Our findings yield further insight into the process of combining clinical and research training in addiction medicine, and can be used to inform similar programs elsewhere.

That current and upcoming physicians held positive attitudes towards the training was not surprising, given their self-selection for the training and the study. It is possible that doctors, who participate in specialised training, already have some experience with addiction research and appreciation for the field [45]. Although literature confirmed that attitude and competency gain takes longer to demonstrate, our participants reported positive learning outcomes, such as increased knowledge following the training [13]; however, it is unclear whether combined clinical and research training made them better doctors and whether patient outcomes improved [37, 39]. Future evaluations of clinician-scientist programmes should employ robust study designs and strategies, with a focus on outcome analyses [2, 11, 28]. The analyses should assess the impact upon provider behaviour and patient outcomes, as per Kirkpatrick’s model [8].

The supportive learning environment of the Fellowship and the short, structured research projects were seen to be the most beneficial features of the training. This is consistent with previous literature that described a combined didactic and experiential learning approach to clinical-scientist education in addiction [6, 11, 38]. Qualitative meta-syntheses concur with these results, placing further emphasis on the role of mentoring [44]. Research regarding strategies to increase research productivity among early-career investigators in the addictions field is scarce [17]. Several formative experiences influenced research productivity among participants in our study. Some literature suggests a decline in learners’ productivity is common once they enter clinical service, probably due to competing priorities and work-life balance [14, 42]. Our findings suggest that having to balance one’s competing priorities as a physician, scientist, teacher, parent or spouse can both hinder and foster research productivity. The increasing demands limit the number of projects that clinical-scientists can work on, but they force them to make the best use of their time. Strategies, such as mentorship, adequate reimbursement, and maintenance of work-life balance can further support the development of physicians as addiction clinician-scientists [23]. More research is required to compare and contrast findings from this training program to other training models, such as the CRITS or the Substance Abuse Research Education and Training (SARET) [26, 50]. While those models aimed to increase trainees’ knowledge, skills and attitudes in addiction medicine, or interest in research, our model capitalized on the immersive learning method and expertise of HIV scientists who mentored the next generation of physician researchers and aimed to increase their productivity. Our trainees also received stipends in addition to their clinical salaries. We designed the programme to be lecture-light and flexible.

Our interviewees suggested several improvements for physician training in addiction research. They unanimously agreed that the training should continue in the future. This requires continued funding for the programme. The programme should be scaled-up [24]. Indeed, the clinician-scientist “species” are thought to be on the verge of extinction [23], and recently announced funding cuts to MD/PhD programmes in Canada further endanger the education of specialists and thus, threaten efforts to improve care and treatment of people with addictions [7]. Training the next generation of physicians in addiction medicine and research can speed up the development and uptake of new treatments [1]. In agreement with previous qualitative research from other disciplines, “learning to account for the social determinants of health” affecting marginalised population is key in this type of training [16, 32]. Although clinician-scientist training alone won’t solve the addiction problem, it is the Archimedean “one firm spot on which to stand,” and has potential to advance the discovery of new therapies and their translation into routine care.

Our research should be interpreted with caution. Our sample was relatively small and comprised of residents, students and staff from a single Canadian programme, which may limit the transferability of our findings. Our study evaluated the learning experiences of trainees at one institution and their views may not necessarily relate to trainees at other institutions, although they do hold insight for other similar training programs. Our participants were not selected randomly, although we invited everyone who was involved in the training and achieved an excellent response rate. We achieved data saturation as recommended for nonprobabilistic sample sizes [15]. It is probable that physicians who seek specialised training are more likely to have positive attitudes towards, and more educational experience with, people who have addiction issues [45]. We recognize that our learning culture may be more fostering or conducive to promoting research to trainees. Nevertheless, the area has traditionally served as an environment where new drug-related training policies and approaches are tested and, if found effective, may be applied elsewhere. With the movement to enhance addiction medicine training within larger U.S. medical schools, future research should examine whether similar efforts to foster research within other clinical training venues produce the same benefit. The insight into experiences of a unique combination of training in addiction medicine and research provided by this qualitative analysis is valuable, and provides a detailed and nuanced understanding of training trajectories and experiences.

## Conclusion

Combining clinical and research training in addiction medicine is feasible and acceptable for current and upcoming doctors; it is not easy, though. Most learners’ experience the training positively. We must understand the experience of addiction physicians in the clinician-scientist track if we want to improve curricula and research productivity, as well as patient outcomes in this field. Evaluation of track record in terms of research productivity needs to be supplemented by an experiential dimension.
